# Orthodontic treatment for disabled children: a survey of parents’ attitudes and overall satisfaction

**DOI:** 10.1186/1472-6831-14-98

**Published:** 2014-08-05

**Authors:** María Teresa Abeleira, Elisabeth Pazos, Isabel Ramos, Mercedes Outumuro, Jacobo Limeres, Juan Seoane-Romero, Marcio Diniz, Pedro Diz

**Affiliations:** 1 Department of Paediatric Dentistry, School of Medicine and Dentistry, Santiago de Compostela University, Santiago de Compostela, Spain; 2 OMEQUI Research Group, Santiago de Compostela University, Santiago de Compostela, Spain; 3 Department of Orthodontics, School of Medicine and Dentistry, Santiago de Compostela University, Santiago de Compostela, Spain; 4 Special Needs Unit, School of Medicine and Dentistry, Santiago de Compostela University, Santiago de Compostela, Spain

**Keywords:** Disabled, Special needs, Orthodontic, Dentistry, Satisfaction

## Abstract

**Background:**

Many patients with disability require orthodontic treatment (OT) to achieve adequate oral function and aesthetic appearance. The cooperation of disabled patients and of their parents is central to the success of OT, as treatment can involve ethical dilemmas. The aim of this study was to analyze the motivation, expectations and overall satisfaction with OT among parents of patients with disabilities.

**Methods:**

The parents of 60 disabled Spanish children with physical, mental and/or sensory impairment undergoing OT were surveyed on attitudes to OT and level of satisfaction with the outcomes. The survey consisted of 23 questions in 4 sections: attitude and adaptation, benefits, adverse effects, and level of satisfaction after completion of OT. A control group formed of the parents of 60 healthy children undergoing OT at the same institution were also surveyed.

**Results:**

Parents of disabled children undergoing OT showed a high level of motivation and they are willing to collaborate in oral hygiene procedures. Adaptation to the removable appliances was poorer in disabled children but adaptation to fixed appliances was excellent. OT can provide a marked improvement in quality of life, social relationships and oral functionality in disabled children.

**Conclusions:**

Among parents of disabled children undergoing OT, the perceived level of overall satisfaction was very high and expectations were often exceeded.

## Background

Orthodontics and Dentofacial Orthopedics is “the dental specialty that includes the diagnosis, prevention, interception and correction of malocclusion, including neuromuscular and skeletal abnormalities of the developing or mature orofacial structures” [1]. The prevalence of severe malocclusion is particularly high among individuals with physical and/or mental disabilities [2,3].

Not only do orthodontic alterations compromise oral function, they also represent an obstacle to the social acceptance of physically and learning disabled persons from an aesthetic point of view [4-6]. It has been estimated that approximately 75% of patients with disability require orthodontic treatment (OT) to achieve and maintain an optimal occlusal relationship necessary to ensure adequate oral function and aesthetic appearance [5,6]. The cooperation of disabled patients and of their parents is central to the success of OT [7], as treatment can involve ethical dilemmas that have been discussed in detail elsewhere [8].

The OT of disabled patients has been examined in a number of case reports and case series in the literature [9-11]. Parents play an important role in the uptake of orthodontic care and are the single most important factor in the motivation for treatment [12]. Our literature search has revealed very few studies that have analyzed the factors conditioning the response to OT and the parents’ attitudes to orthodontic care, and none of those studies has included a control group of non-disabled individuals [13-15].

The aim of our study was to analyze the motivation and expectations of parents regarding the OT of disabled patients, the social implications, the adverse effects of therapy and the level of satisfaction with treatment outcomes.

## Methods

The parents of 60 disabled children (DCh) with physical, mental and/or sensory impairment undergoing OT were surveyed on attitudes to OT and the level of satisfaction with outcomes. All treatments were performed between 2010 and 2013 in the Special Needs Unit of the School of Medicine and Dentistry of the University of Santiago de Compostela in Spain. The mean age of patients was 13.8 ± 2.3 years (range, 9–18 years). All patients lived at home or in an institution and no overnight stays in our unit were required. All patients were able to tolerate dental procedures using only behavior modification techniques; a single session of deep sedation or general anesthesia was needed in only 4 DCh patients for long procedures requiring a high degree of collaboration (taking impressions and bracket adhesion). Patients with severe malocclusions requiring jaw surgery were excluded.

The orthodontic diagnosis was established in each patient based on the following variables: anteroposterior malocclusion (Angle’s classification system), transverse malocclusion, and pre-treatment Peer Assessment Rating (PAR) index. The orthodontic treatment outcome was evaluated by using the difference between pre-treatment and post-treatment PAR index scores (absolute value and percentage), and the PAR nomogram (worse or no different, improved, greatly improved).

The medical diagnoses in the study group were Down’s syndrome (13 cases), mental and/or psychomotor deficiency (12 cases), congenital malformations with craniofacial involvement (10 cases), cerebral palsy (7 cases), autistic spectrum disorders (4 cases), sensory deficiencies (4 cases) and other rare congenital disorders (10 cases).

The survey was based on previously validated questionnaires [13-15], and consisted of 23 questions grouped into 4 sections (Spanish version, see Additional file 1).

1. Attitude and adaptation to OT: Patient attitude and parent capacity to help with oral care during treatment, frequency of daily toothbrushing, level of collaboration with oral hygiene procedures, adaptation to fixed and removable appliances and influence of treatment on activities of daily living.

2. Benefits derived from OT: Improvement in quality of life, social acceptance and integration, importance of aesthetic appearance and other reasons for seeking treatment.

3. Adverse effects associated with OT: Oral lesions, altered oral function, increased salivary secretion and nausea.

4. Level of satisfaction after completion of OT: Satisfaction with the outcome, improved self-image, reaction of family and friends, improvement in daily activities, changes in social life, and willingness to undergo orthodontic treatment again in the future should it be required.

To establish a control group, the parents of 60 healthy children (HCh) undergoing OT at the Orthodontic Unit of the same institution and treated by the same orthodontists were also surveyed. HCh were matched with the DCh group for age, sex, anteroposterior malocclusion and pre-treatment PAR index score. All patients (DCh and HCh) were treated with both removable and fixed appliances.

The statistical analysis of the results was performed using R software, version 2.12.0 (R Development Core Team, Vienna, Austria). Differences between the responses of DCh and HCh parents were analyzed using the Fisher test with significance taken as a *P* value less than 0.05. The Kruskal Wallis test was used to analyse differences in the values of the qualitative variables between patients with different medical diagnoses.

### Ethics and consent

The study protocol was approved by the Ethics Committee of the University of Santiago de Compostela (reference number 2010-1724B). Written informed consent was obtained from the parents or legal guardians, as applicable, of the involved disabled and healthy children undergoing orthodontic treatment . A copy of the written consent is available for review by the Editor of this journal.

## Results

### Orthodontic diagnoses

The anteroposterior malocclusion diagnosis in the DCh group was Class I in 25.3% of cases, Class II in 33.7%, and Class III in the remaining 41%; the malocclusion was due to alterations in the maxilla in 13.2% of cases, alterations in the mandible in 13.2% of cases, and bimaxillary in 73.6%. Transverse occlusal alterations were observed in 49% of the DCh group: unilateral crossbite in 21% and bilateral crossbite in 27.8%. The mean PAR index score was 31.6 ± 7 pre-treatment and 10.4 ± 8.4 post-treatment. The mean difference between pre-treatment and post-treatment PAR was 21.2 ± 5 (69.9 ± 20.1%); after OT, 39.8% of DCh parents considered that there had been a marked improvement, 54.2% that their child had improved, and 6% that treatment had not produced any improvement.

No differences were detected in the distribution of anteroposterior malocclusions or in the pre-treatment PAR index scores between the DCh and the matched HCh.

The medical diagnosis of the patients influenced the prevalence of anteroposterior malocclusions (*P* = 0.02) (Table 1). However, it did not affect the prevalence of transverse malocclusions, the distribution of maxillary, mandibular, or bimaxillary alterations, pre-treatment PAR, post-treatment PAR, difference between pre-treatment and post-treatment PAR, or the PAR nomogram.

**Table 1:** Diagnoses of anteroposterior malocclusion (Angle’s classification system) in disabled children (n = 60)

**Medical diagnoses**	**Class I**	**Class II**	**Class III**
Down’s syndrome	0	1	12
Mental and/or psychomotor deficiency	4	3	5
Congenital malformations with craniofacial involvement	1	5	4
Cerebral palsy	0	5	2
Autistic spectrum disorders	0	2	2
Sensory deficiencies	1	0	3
Other rare congenital disorders	3	3	4

### Attitudes and adaptation

According to parents, 83.3% of DCh were particularly motivated during therapy. A large majority (91.6%) of DCh parents felt fully prepared to perform oral care during OT compared with 60.0% of HCh parents, 40.0% of whom admitted that they were not ready (*P* < 0.001). An increase in daily toothbrushing was detected in 55.0% of DCh, and 66.6% of these patients performed this activity 2 to 3 times a day. A high level of collaboration in daily oral hygiene procedures was reported by 60.0% of the parents of DCh (even prior to OT) in contrast to 1.6% of HCh parents, and 83.3% of HCh showed a poor collaboration in these procedures (*P* < 0.001). Adaptation to wearing removable appliances was poorer in DCh than in HCh (71.4% versus 93.8%, *P* = 0.019), and 11.9% of DCh did not tolerate the appliance. Adaptation to fixed appliances was excellent in both the DCh and the HCh groups (91.6% and 100%, respectively). While 36.6% of DCh parents agreed that no individual phase of OT was more difficult than the other stages, 51% of HCh parents suggested that the fixed appliance phase offered the greatest difficulty (*P* < 0.001). The maintenance of oral hygiene during OT was considered particularly stressful by 46.6% of DCh parents compared with 85% HCh parents (*P* < 0.001). These results are detailed in Table 2.

**Table 2:** Opinions of parents of disabled (n = 60) and healthy children (n = 60) on attitudes and adaptation to appliances during orthodontic treatment

	**Disabled children n (%)**	**Healthy children n (%)**	***P*** **value**
**Patient’s attitude**			1.000
She/he was particularly motivated during therapy	50 (83.3%)	52 (86.6%)	
She/he understood the therapy, but was not motivated	5 (8.3%)	8 (13.3%)	
She/he did not understand the therapy	5 (8.3%)	0 (0%)	
**Parent’s preparation for oral care**			< 0.001
Fully prepared	55 (91.6%)	36 (60.0%)	
Ready to act if necessary	2 (3.3%)	0 (0%)	
Prepared to guide and encourage caregivers	1 (1.6%)	0 (0%)	
Not ready	2 (3.3%)	24 (40.0%)	
**Daily toothbrushing**			0.154
Increased	33 (55.0%)	25 (41.6%)	
Not increased	27 (45.0%)	35 (58.3%)	
**Number of daily toothbrushings before orthodontic treatment**			0.812
Once a day	20 (33.3%)	23 (38.3%)	
2 to 3 times a day	40 (66.6%)	37 (61.6%)	
**Level of collaboration in oral hygiene procedures**			< 0.001
High prior to orthodontic treatment	36 (60.0%)	1 (1.6%)	
High from the start of orthodontic treatment	16 (26.6%)	9 (15.0%)	
Low	8 (13.3%)	50 (83.3%)	
Adaptation to removable appliance*			0.019
She/he did not tolerate the appliance	5 (11.9%)	0 (0%)	
She/he adapted to the appliance after some time	7 (16.6%)	3 (6.1%)	
She/he adapted to the appliance immediately	30 (71.4%)	46 (93.8%)	
**Adaptation to fixed appliance**			0.026
She/he did not tolerate the appliance	2 (3.3%)	0 (0%)	
She/he adapted to the appliance after some time	3 (5.0%)	0 (0%)	
She/he adapted to the appliance immediately	55 (91.6%)	60 (100%)	
**Most** d**ifficult orthodontic phase**			< 0.001
Removable phase	16 (26.6%)	22 (36.6%)	
Fixed phase	16 (26.6%)	30 (50.0%)	
Removable extraoral phase	6 (10.0%)	8 (13.3%)	
None of the above	22 (36.6%)	0 (0%)	
**Aspects found particularly overwhelming**			< 0.001
Insertion of the device each day	5 (8.3%)	9 (15.0%)	
Taking care of treatment	1 (1.6%)	0 (0%)	
Maintenance of oral hygiene	28 (46.6%)	51 (85.0%)	
None of the above	26 (43.3%)	0 (0%)	

*This question was only answered by parents of disabled and healthy children who wore removable appliances (n = 42 and n = 39, respectively).

### Perceived benefits of OT

An improvement in the quality of life of the child after OT was reported by 83.3% of DCh parents compared with 78.3% of HCh parents (not significant). However, while 78.3% of DCh parents considered that OT had improved social acceptance and 71.6% that it had increased social integration, each of these changes was only reported by 55.0% of HCh parents (*P* = 0.016 and *P* = 0.006, respectively). The reason that DCh parents requested OT was to improve dental health in 53.3% and to improve speech in 21.6%, compared to 75.0% and 1.6%, respectively, among HCh parents (*P* = 0.002). These results are detailed in Table 3.

**Table 3:** Benefits of orthodontic treatment as perceived by parents of disabled (n = 60) and healthy children (n = 60)

	**Disabled children n (%)**	**Healthy children n (%)**	***P*** **value**
**You wish to enhance the appearance of the teeth and face**			0.228
A lot	41 (68.3%)	30 (50.0%)	
A little	12 (20.0%)	19 (31.6%)	
Not concerned	7 (11.6%)	11 (18.3%)	
**Improvement in quality of life**			0.536
Yes	50 (83.3%)	47 (78.3%)	
No	4 (6.6%)	6 (10.0%)	
Don’t know	6 (10.0%)	8 (13.3%)	
**Improvement in social acceptance**			0.016
Yes	47 (78.3%)	33 (55.0%)	
No	7 (11.6%)	10 (16.6%)	
Don’t know	6 (10.0%)	17 (28.3%)	
**Improvement in social integration**			0.006
Yes	43 (71.6%)	33 (55.0%)	
No	10 (16.6%)	6 (10.0%)	
Don’t know	7 (11.6%)	21 (35.0%)	
**Other reasons for seeking treatment**			0.002
To improve dental health	32 (53.3%)	45 (75.0%)	
To improve chewing	15 (25.0%)	14 (23.3%)	
To improve speech	13 (21.6%)	1 (1.6%)	

### Adverse effects associated with OT

Oral lesions were detected by 59.9% of DCh parents during OT. In most patients (76.6% of DCh and 85.0% of HCh) the appliances did not alter everyday oral function. An increase in saliva production and/or nausea was observed in 20.0% of DCh compared with 5.0% of HCh (*P* = 0.008). “Alteration of everyday oral function” was more common among patients with the highest post-treatment PAR scores (*P* = 0.04) and those with the largest differences between pre-treatment and post-treatment PAR scores (*P* = 0.03). These results are detailed in Table 4.

**Table 4:** Adverse effects of orthodontic treatment as perceived by parents of disabled (n = 60) and healthy children (n = 60)

	**Disabled children n (%)**	**Healthy children n (%)**	***P*** **value**
**Oral lesions**			0.473
Frequent	5 (8.3%)	0 (0%)	
Occasional	31 (51.6%)	31 (51.6%)	
No	24 (40.0%)	29 (48.3%)	
**Alteration of everyday oral function**			0.179
Frequent	2 (3.3%)	0 (0%)	
Occasional	12 (20.0%)	9 (15.0%)	
No	46 (76.6%)	51 (85.0%)	
**Increased salivary secretion and/or nausea**			0.008
Frequent	3 (5.0%)	0 (0%)	
Occasional	9 (15.0%)	3 (5.0%)	
No	48 (80.0%)	57 (95.0%)	

### Satisfaction and improvements

Satisfaction with the outcomes of OT was indicated by 51.5% of DCh parents and 42.4% of them stated that the results exceeded expectations, compared with 90.0% and 10.0%, respectively, of HCh parents (*P* < 0.001). Results exceeded expectations particularly in patients with the highest pre-treatment PAR scores (*P* = 0.02), and those with highest differences between pre-treatment and post-treatment PAR scores (*P* = 0.03).

Relatives and friends got excited about OT results in 54.5% of DCh patients whereas no reaction was observed in 71.6% of the HCh group (*P* < 0.001). The percentage of relatives who were enthusiastic about OT outcomes was higher among patients with the highest pre-treatment PAR scores (*P* = 0.02), those with greatest differences between pre-treatment and post-treatment PAR scores (*P* < 0.01), and those with a marked improvement on the PAR nomogram (*P* = 0.01).

The number of parents who observed a significant improvement in daily activities and social life after OT was significantly higher among DCh (81.8% and 45.4%) than among HCh (10.0% and 5.0%) (*P* < 0.001 in both groups). Improvement in patient daily activities was most evident among patients with the highest pre-treatment PAR scores (*P* < 0.01), and those with the greatest differences between pre-treatment and post-treatment PAR scores (*P =* 0.02). Improvement in patient’s social life increased in those patients with the greatest differences between pre-treatment and post-treatment PAR scores (*P* < 0.01).

When parents were asked if they would allow their children to undergo similar treatments in the future, if required, 93.9% of DCh parents and 90.0% of HCh parents responded positively.

All these results related to “Satisfaction and improvements” are detailed in Table 5.

**Table 5:** Level of satisfaction and appreciation of improvements by surveyed parents of disabled (n = 33) and healthy children (n = 60), after completion of orthodontic treatment

	**Disabled children n (%)**	**Healthy children n (%)**	***P*** **value**
**Satisfaction with the results**			0.000
Satisfied but with excessive effort	2 (6.0%)	0 (0%)	
Satisfied	17 (51.5%)	54 (90.0%)	
The results exceeded expectations	14 (42.4%)	6 (10.0%)	
**Improvement in patient’s self-image**			0.228
None	3 (9.0%)	3 (5.0%)	
The child is not satisfied with the result	1 (3.0%)	0 (0%)	
The child is pleased with the result	29 (87.8%)	57 (95.0%)	
**Reaction of family and friends**			< 0.001
No reaction	6 (18.1%)	43 (71.6%)	
They encouraged us throughout the treatment	9 (27.2%)	6 (10.0%)	
They got excited	18 (54.5%)	11 (18.3%)	
**Improvement in patient daily activities**			< 0.001
No change	5 (15.1%)	40 (66.6%)	
Progress was significant	1 (3.0%)	14 (23.3%)	
There was a very marked improvement	27(81.8%)	6 (10.0%)	
**Change in patient’s social life**			< 0.001
None	9 (27.2%)	34 (56.6%)	
Slightly improved social life	9 (27.2%)	23 (38.3%)	
Significantly improved social life	15 (45.4%)	3 (5.0%)	
**The child is ready to undergo orthodontic treatment in the future, if needed**			0.711
Yes	31 (93.9%)	54 (90.0%)	
No	2 (6.0)	6 (10.0%)	

## Discussion

In our experience, OT may take longer to complete in DCh patients than in the general population due to the complexity of the malocclusions, the greater number of appointments required, and occasionally due to the temporary withdrawal of appliances (a consequence of traumatic lesions, gingival thickening, or poor oral hygiene). Motivation is a key factor in achieving good cooperation during OT [13]. In HCh undergoing OT it has been suggested that the level of motivation does not increase during the different phases of treatment and that it is conditioned by the presence of discomfort and by the degree of acceptance of the device; furthermore, parents are often significantly more motivated than their children for OT to be performed [16]. Disabled individuals usually receive continuous daily attention from motivated parents, who are willing to do everything possible to increase their child’s well-being [13]. The DCh parents are often therefore willing to become members of the orthodontic team [7]; this occurred in the present series, in which DCh parents had a significantly higher degree of motivation than HCh parents.

Inadequate oral hygiene can be the greatest obstacle to the success of OT [7,14]. Atassi et al. [17] in a series of healthy patients undergoing OT, found that 60% of them had poor oral hygiene, confirming the need to develop oral hygiene maintenance programs for application during OT. In the present series, over 80% of HCh parents stated that the level of collaboration in their child’s oral hygiene was low. Waldman et al. [5] considered that the principal limitations to OT in physically and learning disabled patients were their lack of understanding of the need for good oral hygiene and their reduced ability to perform adequate hygiene techniques. However, in the present study, the frequency of toothbrushing before and during OT was similar in the study and control groups.

A high percentage of HCh report feeling less discomfort with removable orthodontic appliances than with fixed ones [18], although greater collaboration was required from patients during the fixed appliance phase [16]. Apart from rare exceptions that require the use of removable appliances in patients with intellectual disability [14], our findings coincided with the majority of authors who have stated that these patients tolerate fixed multibracket appliances better [6].

Becker et al. [14] suggested that the two problems most frequently detected during OT in DCh were the maintenance of adequate oral hygiene and difficulties in monitoring treatment. In our survey, most HCh parents found maintaining oral hygiene stressful; this was less common among the DCh parents. Parents actively involved in the day-to-day care of a child’s well-being are highly motivated when seeking OT [7], which may explain why more than 40% of surveyed DCh parents did not find any phase of OT an overwhelming problem.

In some published series it has been found that over 90% of HCh undergoing OT and their parents were concerned about aesthetic appearance and that this was the main reason for requesting OT [19]. Al-Sarhad et al. [15] concluded that parents of sensory impaired children (blind and deaf) were aware of their child’s dental aesthetic appearance and that this had a determining role in seeking OT. In physical and learning disabled patients, improved facial appearance has also been reported as the most common reason to request OT [13]. In the present series we did not observe statistically significant differences between the percentage of DCh and HCh parents for whom enhancing the facial appearance of their children was important.

Results of surveys conducted in healthy children undergoing OT revealed that both parents and children felt that an aesthetically pleasing result was important for psychosocial well-being [20]. Improved physical appearance and oral function following OT could increase the quality of life of DCh and promote their social acceptance [13]. This could explain the improvement in social relationships of DCh observed in the present series. However, in previous studies, parents have expressed their difficulty in appreciating these improvements, particularly in patients unable to express their feelings [21].

Becker et al. [13] showed that OT in DCh could not only improve the facial appearance but also masticatory function, speech and drooling control. In a survey performed on Swedish parents of DCh, the improvement in chewing and speech and the reduction of dental trauma were considered the principal benefits of OT [21]. In the present study, parents also requested OT for reasons related to orofacial function, as may be appreciated by the remarkable difference observed in speech improvement in DCh compared with HCh.

It has been shown that OT carries an increased risk of oral mucosal lesions in healthy individuals aged between 6 and 18 years, with gingival inflammation, erosion, ulceration and contusion being the most common findings [22]. In some DCh, such as those with Down’s syndrome, oral ulcers can be the most common complication during OT [11]. In the present series, more than half of HCh parents and 60% of DCh parents declared that their children had oral lesions arising from OT. It has been suggested that adequate oral hygiene instructions and the early treatment of oral lesions are important considerations to increase patient motivation and to complete OT successfully [22]. Pain and discomfort during OT also strongly affect treatment satisfaction [23]. As for the disruption of daily functions during OT, Stewart et al. [18] reported that speech and swallowing disorders appeared in patients wearing removable appliances, and that these persisted in some cases even after 3 months. In the present series, over 75% of DCh parents and 85% of HCh parents confirmed that OT did not cause any alteration in everyday oral functions. However, a significantly higher percentage of DCh than HCh controls presented increased salivary secretion and/or nausea during OT.

In a study conducted on healthy adolescents who had received OT, it was found that only 34% were completely satisfied with results, 62% were relatively satisfied and 4% were dissatisfied [24]. These findings contrast with those described by Becker et al. [13] in a series of DCh in which 100% of parents surveyed said they felt satisfied with results, and 11% even said their expectations had been exceeded. In the present series, over 90% of DCh parents were satisfied, and over 40% stated that OT outcomes had exceeded their expectations; 100% of HCh parents stated they were satisfied with the outcome. These findings in both DCh and HCh may be related to other recently suggested factors conditioning satisfaction with OT outcome, such as quality of care and attention [23]. In a series published by Becker et al. [13], 63% of DCh parents noted positive changes in their child’s oral function after OT. In our series, the perception of improvement in daily activities and in the perceived changes in social life was significantly higher in DCh than in HCh. These improvements will lead to greater self-confidence and satisfaction, and the parents of DCh would therefore allow their children to undergo the same treatment in the future, should it be necessary. Similarly, DCh parents surveyed by Becker et al. [13] would recommend OT to other patients and they would repeat the same procedure should the initial circumstances recur.

This study has certain limitations that must be taken into account, as they may potentially affect the results of the survey. The characteristics of the DCh group meant that their parents answered the questionnaire, and their motivation and expectations may have been exaggerated. The degree of patient collaboration is implicit in the inclusion criteria, as OT is indicated primarily in cooperative patients. The PAR index score had an impact on certain responses, particularly those relating to the degree of satisfaction and appreciation of improvement; as very severe malocclusions, such as those requiring surgical management, were excluded, we do not know if the replies would have been different in patients with higher PAR index scores. Finally, some functional variables, such as the improvement in patient daily activities, were evaluated on a qualitative basis and would benefit from a more objective assessment.

## Conclusions

The results of this study confirm that parents of DCh undergoing OT show a high level of motivation and that they are willing to collaborate in oral hygiene procedures. OT can produce a considerable improvement in quality of life, social relationships and oral function in DCh. The perceived level of satisfaction is often very high and parents of these children stated that they would repeat OT, if needed, in the future.

## Abbreviations

OT: Orthodontic treatment; DCh: Disabled children; HCh: Healthy children.

## Competing interests

The authors report no declarations of interest.

## Authors’ contributions

MTA, EP, JS-R and IR performed the orthodontic treatment. MO and JL designed and applied the survey. MD analyzed data and drafted the manuscript. PD conceptualized the study and drafted the manuscript. All authors provided comments on the original draft and contributed to the development of the final draft. All authors read and approved the final manuscript.

## Pre-publication history

The pre-publication history for this paper can be accessed here:

http://www.biomedcentral.com/1472-6831/14/98/prepub

## Supplementary Material

Additional file 1 Spanish version of the survey. Click here for file
