# Supplementary material for: Orthodontic treatment for disabled children: a survey of parents’ attitudes and overall satisfaction
Source: BMC Oral Health. 2014 Aug 5;14:98. doi: 10.1186/1472-6831-14-98 (PMC4134462; doi:10.1186/1472-6831-14-98)
Supplement: Additional file 1 — Spanish version of the survey. [file 1472-6831-14-98-S1.doc]

CUESTIONARIO DE ORTODONCIA

¿ Quién te refirió o sugirió el tratamiento de ortodoncia?

□ Lo decidimos nosotros, nadie en particular nos lo sugirió.

□ El pediatra/ Médico general.

□ El dentista

□ El trabajador social.

□ Amigos / Familiares.

¿Dónde vive el niño?

□ En casa.

□ Con una familia adoptiva.

□ En una institución.

Si el niño vive en una institución, ¿con que frecuencia va a casa?

□ Cada tarde.

□ Una o dos veces a la semana.

□ Una o dos veces al mes.

¿Cuál es la actitud del niño ante el tratamiento de ortodoncia?

□ No entiende por qué lo estamos haciendo.

□ Es consciente de su problema dental pero no está interesado en el tratamiento.

□ Es consciente del problema dental y está interesado en el tratamiento.

□ Está muy motivado para el tratamiento.

¿Amigos, familiares o vecinos han influido para buscar tratamiento para tu niño?

□ Sí.

□ No.

Cuantas veces se cepillaba al día antes de iniciar el tratamiento:

□ Ninguna.

□ 1 al día.

□ 2 -3 veces al día.

En relación a la higiene oral del niño ¿Cuántas veces se cepilla al día?

□ Ha aumentado el número de cepillados al día desde que inició el tratamiento ortodóncico.

□ No ha cambiado el número de cepillados al día desde que inició el tratamiento ortodóncico.

□ Ha disminuido el número de cepillados al día desde que inició el tratamiento ortodóncico.

¿Colabora en el cepillado diario del niño? (como mínimo en alguno de los cepillados) :

□ Siempre, incluso antes del tratamiento de ortodoncia.

□ Sí, desde que inició el tratamiento de ortodoncia.

□ No, nunca ayuda ni antes ni después de iniciar el tratamiento ortodóncico.

El tratamiento de ortodoncia exige tu completa colaboración. ¿En que grado estás preparado para ayudar en los cuidados del niño en casa durante el tratamiento (cepillado de los dientes, cuidado y mantenimiento de los aparatos, y otras funciones relacionadas)?

□ Preparado completamente para tomar responsabilidad en el cuidado y mantenimiento de los dientes y del aparato.

□ Preparado para viajar a la institución o casa de acogida para cumplir estas funciones.

□ Preparado para guiar y estimular al personal de la institución para cumplir estas funciones.

□ No está preparado.

¿Cree que arreglar los dientes puede hacer al niño más atractivo y aceptado socialmente, y así mejorar su calidad de vida?

□ Sí.

□ No.

□ No sé.

¿Cree que el tratamiento de ortodoncia puede mejorar su integración en la sociedad?

□ Sí.

□ No.

□ No sé.

¿Cree que el corregir los dientes de su niño puede influir positivamente en su propia aceptación social?

□ Sí.

□ No.

□ No sé.

Realzar la apariencia de los dientes y la cara, le preocupa:

□ Mucho.

□ Poco.
 □ No le preocupa.

¿Hay otra razón que motivó a buscar el tratamiento ortodóntico, además de realzar la apariencia de los dientes y de la cara?

□ Mejorar la salud dental.

□ Mejorar la masticación.

□ Mejorar el habla.

□ Elimina el babeo.

Después del primer día con el aparato de ortodoncia:

□ Estamos sorprendidos y aliviados por la rápida adaptación al aparato.

□ Al principio había dificultades en el manejo del aparato, que se superaron en poco tiempo.

□ Las dificultades fueron insuperables.

Si el niño lleva aparatos removibles, responda a lo siguiente:

□ No tolera los aparatos removibles, e intenta quitárselos.

□ Al principio intentaba quitárselos pero se adaptó en poco tiempo.

□ Consiguió usar los aparatos rápidamente, y nunca intentó quitárselos.

Si el niño lleva aparato fijo, responda a lo siguiente:

□ Trata de desplazarlo en un intento de quitárselo de los dientes.

□ Esta molesto, e inicialmente pide que se lo quiten.

□ Se adaptó al aparato en poco tiempo.

Aparecen heridas en las encías, mejillas, paladar, etc., que le impidan comer.

□ Sí, con mucha frecuencia.

□ Sí, a veces.

□ No, nunca.

El aparato le interfiere al hablar, respirar o tragar.

□ Sí.

□ No.

□ A veces.

Ha notado que han aumentado la saliva o las náuseas desde que se le puso el aparato.

□ Sí.

□ No.

□ A veces.

¿Cómo describirías su conformidad durante el tratamiento?

□ Aumentó durante el tratamiento.

□ No ha cambiado desde el inicio hasta el final del tratamiento.

□ Disminuyó durante el tratamiento.

¿Cuánto dificulta la vida cotidiana de los familiares?

□ Las responsabilidades añadidas son excesivas.

□ Las responsabilidades añadidas son soportables.

□ Las responsabilidades no alteran nuestra vida cotidiana.

¿Cuál de las siguientes cuestiones le ofreció mayor dificultad?

□ El poner el aparato día a día y su accesorios.

□ Dificultades para ocuparse del tratamiento.

□ Mantenimiento de una adecuada higiene oral.

¿Qué fase del tratamiento presentó mayor dificultad?

□ Usando aparatología removible.

□ Usando aparatología fija.

□ Usando aparatología removible extraoral.

CUESTIONARIO DE ORTODONCIA AL FINALIZAR EL TRATAMIENTO

¿Los resultados del tratamiento fueron satisfactorios?

1. Insatisfecho.
2. Satisfecho, pero siente que el esfuerzo excedió el valor de los resultados.
3. Satisfecho.
4. Los resultados del tratamiento excedieron nuestras expectativas.

¿Tú crees que el corregir sus dientes mejoró su autoimagen?

1. No, el niño ignora la mejoría.
2. El niño no está satisfecho con los resultados de los tratamientos.
3. El niño ve mejoría y ha aumentado su confianza.

¿Cómo reaccionaron los amigos, familiares y vecinos al hecho de que el niño haya sido tratado?

1. No hay reacción obvia.
2. Ellos nos estimularon a lo largo del proceso.
3. Ellos estaban entusiasmados con los resultados del tratamiento.

¿Tu sientes que el tratamiento de ortodoncia ha mejorado las funciones diarias del niño?

1. No hay cambio.
2. El progreso no fue significativo.
3. Hubo una importante mejoría.

¿Sientes que el tratamiento cambió su vida social?

1. No, el tratamiento no tuvo influencia en sus relaciones sociales.
2. El tratamiento mejoró ligeramente su vida social.
3. El tratamiento mejoró su vida social considerablemente.

¿ Someterías al niño al mismo tratamiento de nuevo?

1. Sí.
2. No

¿ Recomendarías el tratamiento de ortodoncia a otros niños discapacitados, si fuera necesario dicho procedimiento?

1. Sí.
2. No.
